# Functions of the Zinc-Sensing Receptor GPR39 in Regulating Intestinal Health in Animals

**DOI:** 10.3390/ijms232012133

**Published:** 2022-10-12

**Authors:** Pengpeng Xia, Li Yan, Xingduo Ji, Yunping Wu, Siqi Lian, Guoqiang Zhu

**Affiliations:** 1 College of Veterinary Medicine (Institute of Comparative Medicine), Yangzhou University, Yangzhou 225009, China; 2 Jiangsu Co-Innovation Center for Prevention and Control of Important Animal Infectious Diseases and Zoonoses, Yangzhou 225009, China; 3 Joint International Research Laboratory of Prevention and Control of Important Animal Infectious Diseases and Zoonotic Diseases of China, Yangzhou University, Yangzhou 225009, China; 4 Joint International Research Laboratory of Agriculture and Agri-Product Safety of Ministry of Education of China, Yangzhou University, Yangzhou 225009, China

**Keywords:** zinc, G protein-coupled receptor 39, signal transduction, intestinal health

## Abstract

G protein-coupled receptor 39 (GPR39) is a zinc-sensing receptor (ZnR) that can sense changes in extracellular Zn^2+^, mediate Zn^2+^ signal transmission, and participate in the regulation of numerous physiological activities in living organisms. For example, GPR39 activates the extracellular signal-regulated kinase/mitogen-activated protein kinase (ERK/MAPK) and phosphatidylinositol3-kinase/protein kinase B (PI3K/AKT) signaling pathways upon Zn^2+^ stimulation, enhances the proliferation and differentiation of colonic cells, and regulates ion transport, as well as exerting other functions. In recent years, with the increased attention to animal gut health issues and the intensive research on GPR39, GPR39 has become a potential target for regulating animal intestinal health. On the one hand, GPR39 is involved in regulating ion transport in the animal intestine, mediating the Cl^−^ efflux by activating the K^+^/Cl^−^ synergistic protein transporter, and relieving diarrhea symptoms. On the other hand, GPR39 can maintain the homeostasis of the animal intestine, promoting pH restoration in colonic cells, regulating gastric acid secretion, and facilitating nutrient absorption. In addition, GPR39 can affect the expression of tight junction proteins in intestinal epithelial cells, improving the barrier function of the animal intestinal mucosa, and maintaining the integrity of the intestine. This review summarizes the structure and signaling transduction processes involving GPR39 and the effect of GPR39 on the regulation of intestinal health in animals, with the aim of further highlighting the role of GPR39 in regulating animal intestinal health and providing new directions and ideas for studying the prevention and treatment of animal intestinal diseases.

## 1. Introduction

G protein-coupled receptors (GPCRs) are the largest family of cell surface receptors in mammals. They participate in the physiological activities of the body by transmitting a variety of extracellular signals such as hormones, neurotransmitters, and odors. At present, this protein family has become the most important drug target library and is targeted by treatments for many diseases [1,2]. This family consists of six groups, namely family A (rhodopsin-like receptors); family B (secretin-like receptors); family C (metabotropic glutamate); family D (fungal mating pheromone receptors); family E (cyclic AMP receptors); and family F (frizzled/smoothened), and family A is the largest group of GPCRs [3]. Among them, G protein-coupled receptor 39 (GPR39) is a typical representative member of family A.

In 1997, Mckee et al. [4] first isolated the cDNA for *GPR39* and *GPR38* from the human brain and preliminarily confirmed that GPR39 and GPR38 were related to ghrelin and neurotensin receptors. Subsequently, GPR38 has been demonstrated to be a homologous receptor for motilin and can regulate gastrointestinal motility [5]. Zhang et al. [6] showed that obestatin (a peptide derived from the precursor of auxin) is the endogenous ligand of GPR39 and has a high affinity with GPR39. However, other researchers did not make similar observations in follow-up experiments. Further studies found that obestatin is not an endogenous ligand of GPR39, but rather zinc ions (Zn^2+^) are the real ligands of GPR39, and they bind to GPR39 predominantly through two histidine residues, His17 (H17), His19 (H19), and aspartic acid residue Asp313 (D313) [7,8,9,10,11]. The GPR39 expressed in fish does not seem to bind to Zn^2+^, indicating that GPR39 may have another endogenous ligand in fish, and this endogenous ligand may also exist in other vertebrates [12]. Due to its ability to interact with a variety of proteins, Zn^2+^ may also be an enhancer and co-activator of other endogenous ligands, and compared with Zn^2+^ stimulation alone, the simultaneous use of Zn^2+^ and the GPR39 activator TC-G1008 was found to further enhance the activity of GPR39. To date, Zn^2+^ is still the only verified endogenous ligand of GPR39.

Intestinal health is crucial for the maintenance of homeostasis in animals [13]; however, intestinal infection continues to be one of the main dangers to animal health. For example, enterotoxigenic *Escherichia coli* (ETEC) F4ac is an important pathogen causing diarrhea in young animals, which causes a high incidence of diarrhea in newborn and weaned piglets and can cause huge economic losses to the pig industry [14]. Zinc is an essential trace element in many organisms. Studies have shown that zinc deficiency or the concentration of Zn^2+^ cannot meet cellular needs, leading to zinc homeostasis and intracellular signal pathway imbalance, affecting animal health [15]. Sargeant et al. [16] found that supplementation of Zn^2+^ can regulate the inflammatory response caused by ETEC infection in porcine intestinal epithelial cells (IPEC-J2), allowing them to maintain normal intestinal function. The mechanism of how zinc affects gastrointestinal health in animals, however, is unfortunately still unclear. The zinc-sensing receptor GPR39 has been shown to exist widely in neurons, colon cells, keratinocytes, pancreatic cells, prostate cancer cells, salivary gland cells, and bone cells. It can sense the concentration of extracellular Zn^2+^ and exert physiological activity [17]. For example, within the physiological concentration range of the digestive tract, extracellular Zn^2+^ can activate the GPR39 of colonic epithelial cells, which can be involved in controlling the growth and differentiation of colonic cells [18,19]. Under the condition of ETEC F4ac infection, GPR39 can inhibit the apoptosis of IPEC-J2 cells to some extent [20]. Based on an understanding of the physiological function of Zn^2+^, this paper briefly introduces the structure and signal transduction process of GPR39 and summarizes the zinc-dependent physiological functions of GPR39 in the animal intestine, in order to better understand the role of GPR39 in animal intestinal health, and lays a foundation for exploring its potential application value.

## 2. The Structure of GPR39

GPR39 is mainly composed of three parts. The extracellular domain consists of the amino-terminus (N-terminus) and three extracellular loops (ECL1-3) of the peptide chain, which are mostly responsible for recognizing various signal molecules, binding corresponding ligands, and initiating downstream signal transduction. The extracellular domain of the GPR39 protein contains two disulfide bonds: the first is between cysteine Cys11 (N-terminal tail) and Cys191 (ECL2), and the second is between Cys108 (the extracellular end of transmembrane segment Ⅲ, TM-Ⅲ, ECL-1) and Cys210 (ECL2). Except for the absence of the first disulfide bond in the GPR39 sequence of fish, the two disulfide bonds are conserved in the GPR39 sequences from other vertebrates [21]. It should be noted that the first disulfide bond is not necessary for GPR39 expression and activation but plays an important role in inhibiting GPR39-mediated signal transduction. This disulfide bond mutation also can increase the ability of the GPR39 agonist (Zn^2+^) to activate GPR39. The second disulfide bond is necessary for the correct expression of GPR39 on the cell surface and the activation of GPR39 that is mediated by agonists. This disulfide bond mutation can greatly reduce the expression of GPR39 protein on the cell surface and eliminate the increase in inositol phosphate caused by agonist-induced GPR39 activation [10,21].

The main part of the GPR39 protein structure comprises seven α-helix transmembrane regions, which can not only stabilize the GPR39 structure but also transmit extracellular signals to the cell. The intracellular region of GPR39 consists of three intracellular rings (intracellular loops) and a carboxyl-terminus (C-terminus) region, which can bind signal molecules and transmit intracellular signals [22,23]. The C-terminus of the GPR39 protein also contains two cysteine residues (Cys360 and Cys361), which may be palmitoylated sites, but only Cys361 is conserved in the GPR39 of all vertebrates, while Cys360 does not exist in fish and some mammals and birds [10,21].

## 3. Signal Transduction via GPR39

**Figure 1:**
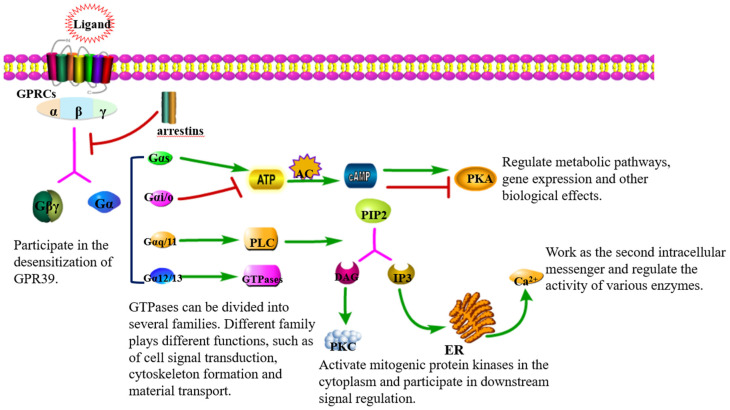
Signal transduction of GPCRs [17,18,25,26,29]. Upon stimulation, GPCRs not only couple to regulatory proteins, such as arrestins, but also trigger the activation of different G protein subtypes including Gαs, Gαi/o, Gαq/11, and Gα12/13, which participate in the signal transduction pathway and mediate unique physiological or pathological functions. An arrow between two elements means that the side being pointed at is activated. Conversely, the symbol (├) represents an inhibiting effect. Abbreviation. ATP: adenosine triphosphate, AC: adenylate cyclase, cAMP: cyclic adenosine monophosphate, PKA: protein kinase A, PLC: phospholipase C, PIP2: phosphatidylinositol-tol-4,5-bisphosphate, DAG: diacylglycerol, PKC: protein kinase C, IP3: 1,4,5-inositol triphosphate, ER: endoplasmic reticulum.

GPCRs play an important role in cellular signal transduction in that they transport extracellular signals from cell membranes to inner cells through guanosine monophosphate binding proteins (G proteins) and participate in important physiological processes (Figure 1). G proteins belong to the GTP enzyme family, which is composed of three protein subunits, namely α subunit, β subunit, and γ subunit, and in which the β and γ subunits can form a stable dimer complex called the β γ subunit. Once an agonist binds to GPCR, the activation of heterotrimer G protein is promoted by the conformational change of the receptor, and then the α subunit and β γ subunit complex are dissociated, both of which can freely activate downstream effectors [24,25]. Gα subunits can be divided into four families: Gαs, Gαi/o, Gαq/11, and Gα12/13, which participate in different signal transduction pathways: (1) Gαs and Gαi/o can activate or inhibit adenylate cyclase (AC) and convert adenosine triphosphate (ATP) into cyclic adenosine monophosphate (cAMP), thus, directly activating or inhibiting protein kinase A (PKA). (2) Phospholipase C (PLC) can be stimulated by Gαq/11, and then phosphatidylinositol-4,5-bisphosphate (PIP2) will be hydrolyzed into diacylglycerol (DAG) and 1,4,5-inositol triphosphate (IP3). DAG is a second messenger that activates protein kinase C, while IP3 induces calcium release from the endoplasmic reticulum (ER), which acts as the second messenger to regulate the corresponding extracellular signal-regulated kinase/mitogen-activated protein kinase (ERK/MAPK) and phosphatidylinositol3-kinase (PI3K)/protein kinase B (AKT)/mammalian target of rapamycin (mTOR) signal pathways. (3) Gα12/13 family can induce serum response element (SRE)-mediated transcription through PI3K and Ras homolog family member A (RhoA) [25,26,27]. Then, the overexpression of GPR39 causes an increased secretion of the cytoprotective pigment epithelium-derived growth factor (PEDF) and other factors and protects against glutamate toxicity, ER stress, and Bax-mediated cell death in a Gα13/RhoA/SRE dependent manner [28].

The activation of GPR39 by Zn^2+^ can increase the concentration of Ca^2+^ and activate the ERK/MAPK and PI3K/AKT signal pathways [17,18]; these two signal pathways play an important role in cell survival and proliferation. The activation of ZnR/GPR39 in keratinocytes, colon cells, and prostate cancer cells can increase the phosphorylation levels of ERK and AKT, thereby promoting cell growth. For example, in androgen-insensitive prostate cancer cell lines, Zn^2+^-activating GPR39 can increase the expression and phosphorylation of AKT and activate the PI3K signal pathway. Consistent with this, when ZnR/GPR39 is desensitized, the phosphorylation of Zn^2+^-dependent ERK1/2 in prostate cancer cells was shown to decrease [30]. In keratinocytes, the activation of ZnR/GPR39 triggers the phosphorylation of MAPK, which increases the scratch closure rate of the skin [31]. After GPR39 is activated, it can promote downstream diverse signal pathways and exert biological functions in various tissues and cells, and whether this protein is activated or not is essential for its normal physiological function.

Like other members of the Ghrelin receptor family, GPR39 has relatively high ligand-independent constitutive activity [32] to induce the activation of Gαq and Gα12/13 and activate the downstream signal pathway [33]. It has been reported that this constitutive activity cannot induce cAMP production, indicating that Gαs is not involved in the activation of downstream signaling [11]. However, Zhang et al. [33,34] assessed the signal pathways activated by GPR39 in different species of spinal animals, and studies have demonstrated that the constitutive signals from GPR39 are different in different vertebrates. GPR39 from humans, chickens, and frogs can activate the Gαq and Gα12 signal pathways, while the GPR39 of zebrafish can activate Gαq, Gα12, and Gαs in a ligand-independent form, and the H296^7.35^ site of zebrafish is the key site of the Gαs signal pathway. At the same time, in the Gαq and Gα12 signal pathways, the constitutive activity of human and zebrafish GPR39 was found to be stronger than that of frog and chicken GPR39. In vertebrate animals, GPR39 has adapted in different ways, resulting in different constitutive activities. Although the physiological roles of GPR39’s constitutive activities are not clear, it is worth noting that due to the existence of these constitutive activities, a change in GPR39 expression may also directly affect downstream signal transmission.

The activation of GPCRs not only triggers the activation of G proteins but also protects against uncontrolled activation by weakening the receptor response associated with other cellular activities, thus, maintaining the homeostasis of the body. In most cases, the first step in weakening the receptor response is mediated by the receptor-specific amino acid phosphorylation of GPCRs by GPCR kinases, especially serine and threonine at the carboxyl terminal of the receptor [35]. After that, arrestins bind to phosphorylated GPCRs, resulting in the uncoupling of GPCRs from G proteins, terminating the initiation of G protein-dependent signal transduction on the cell surface. In addition, arrestins can promote the internalization of GPCRs and act as a molecular scaffold to recruit signal molecules to internalize GPCRs in the interbody, thus, activating signal cascades that are independent of G proteins. When an agonist activates GPCRs, the internalized GPCRs can be recycled back to the cell surface to participate in the next round of signal transduction, in which a portion of GPCRs will be destroyed by lysosomal hydrolysis [25,26]. However, due to the activity of this receptor-specific amino acid phosphorylation by GPR39, heterologously expressed GPR39 in human embryonic kidney cells was found not to induce β-arrestin2 recruitment, even when GPR39 had a high level of phosphorylation. At the same time, co-incubation with Zn^2+^ also led to a decrease in the phosphorylation level of GPR39 [26]. This may have been due to the desensitization of GPR39, which is mediated by Zn^2+^ via Gα12/13 and RhoA [29].

## 4. The Zinc-Dependent Physiological Functions of GPR39

Zinc is the second most concentrated of trace elements in organisms; exists in various organs, tissues, and body fluids; and participates in many aspects of life activities, such as DNA replication, transcription, protein synthesis, cell proliferation, apoptosis, and signal transduction [15]. Intracellular zinc usually exists in two forms. Most zinc binds to metalloenzymes or zinc finger-binding proteins to form a tightly bound Zn^2+^ pool [36], while a small amount of zinc exists in a free or loosely bound state. Studies have shown that when the homeostasis of Zn^2+^ in the body is disturbed, it will cause different degrees of health problems. Zinc deficiency can lead to growth retardation, loss of appetite, impaired immunity, enhanced oxidative stress, skin damage, delayed wound healing, and diminished reproductive ability, while long-term zinc deficiency may lead to immune defects, secondary pathogenic microorganism co-infection, and/or fatal outcomes [15].

As a zinc-sensing receptor (ZnR), GPR39 can be activated by Zn^2+^ and is widely expressed in the gastrointestinal tract, skin, pancreas, salivary glands, heart, testis, bone and cartilage, and sperm cells [37]. Therefore, GPR39 depends on Zn^2+^ to play a physiological regulatory role in different tissues (see Table 1 for details). Nevertheless, the bioactivity of the splice variant GPR39-1b is different from canonical GPR39, which lacks an extracellular loop and carboxyl tail, is expressed in the central nervous and gastrointestinal systems, and shows no response to Zn^2+^ stimulation. It has been reported that GPR39-1b can form a dimer with neurotensin receptor 1 (NTSR1) and negatively regulates its function, and this response is not closely related to zinc or uses GPR39-1b as a functional receptor of Zn^2+^. However, GPR39-1b can regulate the function of GPR39 in a concentration-specific manner, and with its low expression level, GPR39-1b promotes the transport of GPR39 to the plasma membrane through dimerization [38,39].

## 5. GPR39 Depends on Zn^2+^ to Regulate Animal Intestinal Health

Zinc is an essential micronutrient that can affect animal intestinal health. Studies have found that zinc deficiency can lead to an increase in the incidence of diarrhea, and supplementation with Zn^2+^ can effectively prevent or attenuate diarrhea [51,52]. For example, dietary supplementation with Zn^2+^ could alleviate colonic focal leakage induced by *Escherichia coli* α-hemolysin in mice [53], and attenuate intestinal mucosal barrier damage in broilers during *Salmonella* Typhimurium (*S.* Typhimurium) infection [54]. Although dietary supplementation with Zn^2+^ can improve intestinal health, Zn^2+^ can interact with a variety of proteins and regulate the activity of these proteins, such as metallothionein and the ZIP family [55]. Changes in Zn^2+^ concentration can affect the function of a variety of proteins, resulting in poor targeting and therapeutic effects in the treatment of intestinal diseases. GPR39 has a high level of expression in the gastrointestinal tract and is an important regulatory factor in the gastrointestinal tract. After being activated by Zn^2+^, ZnR/GPR39 can participate in the regulation of cellular functions through signal transduction [12]. Therefore, the following section aims to clarify the role of GPR39 in animal intestinal health from the functions of GPR39 in regulating intestinal ion transport, maintaining intestinal homeostasis, and affecting the expression of tight junction proteins in intestinal epithelial cells.

### 5.1. GPR39 Regulates Intestinal Ion Transport in Animals

As a supplement to the treatment of diarrhea, Zn^2+^ can effectively reduce the duration and severity of diarrhea, but it is not clear whether it only counteracts nutritional deficiencies, or whether it can directly regulate solute absorption. Previous studies have shown that GPR39 may play a specific role in regulating intestinal homeostasis. During diarrhea, Zn^2+^ regulates intestinal water salt balance through GPR39, reducing fluid loss [19], and regulating the transport of Na^+^, K^+^, and Cl^−^. This response is zinc specific and could not be triggered by Mn^2+^, Cu^2+^ or Fe^2+^ [17,19,56]. GPR39 can also mediate the Cl^−^ efflux and plays a role in relieving diarrhea symptoms by activating K^+^/Cl^−^ cotransporter (KCC1). In primary colonic cells and human colorectal adenocarcinoma-derived Caco-2 cells, ZnR/GPR39 enhances Cl^−^ transport by upregulating KCC1 [19]. Sunuwar et al. [19] found that when infected with cholera toxin, GPR39^−/−^ mice had more severe diarrhea than wild-type mice, which may have been due to a decrease in KCC1-dependent Cl^−^ transport in the intestine, resulting in increased intestinal fluid secretion. To sum up, GPR39 plays a role in the treatment of diarrhea by activating KCC1 and affecting Cl^−^ transport. Although the WHO recommends Zn^2+^ as an important supplement for the treatment of diarrhea [57], ZnR/GPR39, as a new specific target, may be more effective for regulating gastrointestinal ion transport and treating diarrhea.

### 5.2. GPR39 Maintains Intestinal Homeostasis

To our knowledge, pH homeostasis is necessary for colon cell survival. In a physiological state, animal colon cells are constantly faced with pH challenges because indigestible carbohydrates continue to reduce pH in the intestinal environment, while proteins increase pH [12]. After cytoplasmic acidification, the widely expressed Na^+^/H^+^ exchanger (NHE) is upregulated, which can induce cell pH recovery [58]. In colon cells, GPR39 induces the activation of NHE, which promotes the recovery of pH in colon cells [18]. In addition, D313, one of the Zn^2+^ binding sites of GPR39, is an essential residue for the GPR39 pH sensor. Cohen et al. [59] found that when HT-29 cells were in a pH 7.4 environment, the GPR39-dependent Ca^2+^ response was the highest, while in the pH 7.7 and pH 7.1 environment, the GPR39-dependent Ca^2+^ response decreased by 50% and 62%, respectively. Moreover, an acidic pH of 6.5 resulted in the GPR39-dependent Ca^2+^ response being completely abrogated, while the GPR39-dependent Na^+^/H^+^ exchange activity and the activation of the ERK1/2 or AKT pathways were inhibited. The pH dependence of GPR39 was not eliminated even if H17 or H19 was replaced by alanine (Ala, A). However, when D (D313A) was replaced by A, the GPR39-dependent Ca^2+^ response was similar to the GPR39-dependent Ca^2+^ response triggered by pH 7.4 or 6.5, in which the ERK1/2 and AKT pathways were activated and Na^+^/H^+^ exchange activity was upregulated, and the pH sensitivity of this receptor was restored when the D313A mutant was mutated back to its original residue (A313D) [17,19,59]. Therefore, changes in extracellular pH can directly affect and regulate GPR39 signals.

GPR39 is also an important regulator of gastrointestinal absorption and gastric acid secretion. Moechars et al. [60] found that the gastrointestinal emptying rate and gastric acid secretion of wild-type mice were slower than GPR39^−/−^ mice, which indicated that the activation of GPR39 may slow down gastrointestinal motility and prolong the retention time of nutrients in the gastrointestinal tract, which is beneficial for the absorption of nutrients to some extent. In addition, the activation of GPR39 increases the secretion of somatostatin in primary gastric mucosal cells, which can regulate the gastric acid secretion mediated by gastric parietal cells, but this response did not occur in GPR39^−/−^ cells [61]. In colon cancer cell lines, the activation of the ZnR/GPR39-dependent MAPK and PI3K pathways increases the expression of anti-apoptotic proteins (such as clusterin), thus, attenuating butyric acid-induced apoptosis [62]. In summary, GPR39 plays a role in maintaining intestinal homeostasis by promoting pH recovery in colon cells, regulating gastric acid secretion, and promoting nutrient absorption. In addition, during the recovery from colitis induced by dextran sodium sulfate (DSS), cell proliferation increased in GPR39-expressing mice, but no similar phenomenon was observed in GPR39-deficient mice [63].

### 5.3. GPR39 Affects the Expression of Tight Junction Proteins in the Intestine

The mammalian absorption of Zn^2+^ mostly occurs in the distal portion of the small intestine. Intestinal cells regulate and maintain intracellular Zn^2+^ concentrations mainly through the absorption of exogenous zinc and the efflux of endogenous zinc. Cells up-regulate the mRNA expression of *ZIP1-ZIP14*, belong to the Zrt, Irt-like protein/SLC39 family, mediate the influx and redistribution of Zn^2+^ to increase the absorption of Zn^2+^, and then upregulate the expression of *Divalent Metal Transporter1* (*DMT1*) mRNA in later stages, and cooperate with ZIP to maintain the stability of cell Zn^2+^ concentration. When the concentration of Zn^2+^ is high, the mRNA expression of *metallothionein 1* (*MT1*) and the zinc transporter *ZnT1-ZnT10* (ZnT/SLC30 family) was found to be upregulated, which increases the binding of Zn^2+^ and promotes the efflux of Zn^2+^ [64]. The intestinal mucosal barrier function is crucial to maintain animal health, prevent intestinal tissue injury, and ensure adequate dietary nutrition for the body. The intestinal epithelial tight junction is an important component of the intestinal mucosal barrier and is composed of several unique proteins, including occludin, claudins, and Zos (ZO-1, ZO-2, and ZO-3) [65]. Studies have shown that zinc transporters can not only maintain intracellular Zn^2+^ concentrations, but also regulate the dynamic balance of intestinal epithelial cells. Additionally, ZIP4 contributes to the differentiation and maintenance of Paneth cells, ZIP7 can promote the proliferation of crypt cells, and ZIP14 protects the structure of intestinal epithelial cells through stabilizing tight junctions (such as the phosphorylation of occludin) [64,66,67,68]. An imbalance of Zn^2+^ homeostasis was found in the intestinal cells of ZIP7- and ZIP14-deficient mice. The deletion of ZIP7 can also trigger ER stress in proliferative progenitor cells, resulting in a large number of cell deaths. The deletion of ZIP14 impairs the integrity of the intestinal epithelial barrier by reducing the expression of ZO-1 and claudin-1 proteins [66,67].

The activity and expression regulation of zinc transporters in intestinal epithelial cells are also closely related to ZnR/GPR39 [17]. Shao et al. [69] showed that in *S.* Typhimurium-infected Caco-2 cells, Zn^2+^ maintains the expression of ZO-1, occludin, E-cadherin, and the downstream signal molecule protein kinase C ζ by activating GPR39. Consistent with this, compared with wild-type mice, GPR39 knockout mice had low levels of ZO-1 and occludin expression in the colon and were highly susceptible to DSS-induced colitis [70]. These results suggest that the absence of GPR39 damages the barrier function of the intestinal tract. After GPR39 activation, the AMPK pathway is activated through the calcium/calmodulin-dependent protein kinase kinase (CaMKK)-β pathway, and then improves the intestinal barrier [71]. Therefore, the Zn^2+^-GPR39 axis can regulate the tight junction proteins in intestinal epithelial cells, which means that GPR39 can also improve the barrier function of the intestinal mucosa and maintain intestinal integrity.

## 6. Conclusions

ZnR/GPR39 is widely expressed in the gastrointestinal tract and plays an important role in regulating the intestinal health of animals. This protein affects the treatment of diarrhea by regulating intestinal ion transport, and it also can promote the recovery of pH in colon cells, regulating gastric acid secretion and promoting nutrient absorption, as well as the intestinal mucosal barrier. However, at present, research on GPR39 has mainly focused on humans and mice, and mice are the most used animal model. In the veterinary field, little attention has been paid to this protein, and it has not been reported in pigs, other livestock, or poultry animals.

Although Zn is an essential trace element in animals and is closely related to the biological processes of the body, supplementation with Zn^2+^ plays a specific role in regulating animal intestinal health. Zn^2+^ can interact with a variety of proteins, but it is not an ideal treatment strategy for application to intestinal diseases because of poor targeting and difficulty in controlling dosages. In recent years, due to developments in structural biology, pharmacology, research models, and the increasing attention being paid to animal intestinal health problems, studies on GPR39 crystal structures and Zn^2+^-mediated GPR39 signal transduction and their role in gastrointestinal health have become increasingly detailed. Therefore, using GPR39 as a target to investigate the role of Zn^2+^ in the animal intestinal tract will provide new directions and ideas for the prevention and treatment of animal intestinal diseases.

## Figures and Tables

**Table 1:** Physiological function of GPR39 in the body.

Affected Parts	Physiological Function	References
Cerebrum	Protects neurons from cortisol injury, inhibits the expression of pro-apoptotic proteins, and upregulates the expression of anti-apoptotic proteins.	Mo et al. [40]
Submandibular	Regulates saliva secretion in human submandibular gland cells.	Kim et al. [41]
Adipose tissue	Regulates adipose tissue metabolism, especially lipolysis, and regulates the function of lipases, such as hormone-sensitive lipase and adipose triglyceride lipase.	Petersen et al. [42]
Pancreas	Controls the pancreatic duodenal homeobox-1 to regulate islet development and differentiation; regulates pancreatic secretion and glucose homeostasis.	Holst et al. [43], Moran et al. [44]
Heart	Regulates the Adenosine 5’-monophosphate (AMP)-activated protein kinase (AMPK)-mTOR-S6K1 signal pathway to promote myocardial hypertrophy (a target for the therapy of cardiac hypertrophy), and to improve human aortic valve calcification through the ERK1/2 signaling pathway.	Liao et al. [45], Chen et al. [46]
Vascular endothelium	Regulates vascular endothelial cell activity.	Zhu et al. [47]
Skin	Promotes the proliferation and differentiation of epithelial cells and is beneficial for wound healing.	Nishida et al. [48]
Reproductive system	Mediates acrosomal exocytosis of bovine spermatozoa and overactivation of human spermatozoa.	Allouche-Fitoussi et al. [49]
Skeleton	Regulates collagen synthesis and deposition in osteoblasts.	Jovanovic et al. [50]

## Data Availability

All datasets generated for this study are included in the article.
